# The intensity of the transcriptional response varies across infection with distinct viral strains in an insect host

**DOI:** 10.1186/s12864-025-11365-8

**Published:** 2025-02-21

**Authors:** Allyson M. Ray, Anja Tehel, Jason L. Rasgon, Robert J. Paxton, Christina M. Grozinger

**Affiliations:** 1 Department of Entomology, Pennsylvania State University, University Park, PA USA; 2 Institute for Biology, Martin Luther University Halle-Wittenberg, Halle (Saale), Germany

**Keywords:** Deformed wing virus, Apis mellifera, Warburg effect, Immunometabolism, Strain variation, Transcriptomics

## Abstract

Organisms respond to infectious agents through diverse immune strategies, and may need to cater a specific response to distinct pathogen challenges, such as various strains of a virus, to maximize fitness. Deformed wing virus (DWV) is one of the most damaging viruses of honey bees (*Apis mellifera*) across the globe, with variant DWV-B currently expanding at the expense of variant DWV-A. While previous research has characterized general host transcriptomic responses to viral exposure, host responses to different DWV strains have not been fully explored. Here, we performed experimental infections with the two dominant strains of DWV, A and B, as well as a mixed infection, and conducted transcriptomic analyses to compare differences in host molecular response to infection. We confirmed canonical anti-viral response to DWV infection, including upregulation of Toll pathway genes and the antimicrobial peptides abaecin and hymenoptaecin. Furthermore, our results suggest a potential role of aerobic glycolysis during viral infection in honey bees. DWV-A and mixed infections were associated with differential expression of a much larger number of host genes than infection with DWV-B. That DWV-B potentially elicits a reduced host immune response may provide a mechanistic explanation for its higher virulence and global emergence. Overall, this study provides the first evidence for strain-specific immune responses to DWV infection, and integrates these findings into the broader domain of insect immunity and host-pathogen dynamics.

**Supplementary Information:**

The online version contains supplementary material available at 10.1186/s12864-025-11365-8.

## Background

Exposure to parasites and pathogens is a universal experience for all organisms living on Earth [1, 2]. Organisms defend themselves from these threats through suites of immune strategies deployed at different stages of parasite exposure, from disease avoidance behaviors through cellular and humoral responses to infection and trans-generational immune priming [3–5]. Understanding how host immune systems respond to disease threats, such as viral infections, will allow us to develop strategies to combat current and future disease outbreaks and support vulnerable populations globally.

Insect species, including some critical insect pollinators, have been experiencing population declines across the globe in the past decades [6, 7]. The western honey bee (*Apis mellifera*), a critical pollinator species due to its domestication status [8, 9] and generalist pollination services [10], has experienced a marked increase in colony mortality in recent years, particularly in North America and Europe [11]. Multiple factors have been associated with declining bee health [12], including infections with numerous pathogens and parasites [13–15]. Deformed wing virus (DWV), a (+)ssRNA picorna-like virus, is one of the most important viruses of honey bees [16], with infection across multiple age and behavioral states such as nurses and foragers [17] resulting in a range of symptoms, including deformed wings, declines in learning and memory, accelerated maturation and shortened lifespan [18, 19], and, if infection is severe enough, dwindling and death of the whole colony [20]. DWV infection is associated with increased severity when the virus is transmitted by the *Varroa destructor* mite, an ectoparasite that spreads DWV while it feeds on developing and adult honey bees [15, 21–23], and has been proposed as a critical selective pressure on DWV populations [24–26].

With the introduction of the *Varroa* mite vector and global trade expansion, DWV rapidly spread across the globe [27–29]. Currently, two main master variants (i.e. strains) dominate global DWV genetic diversity, *deformed wing virus A* (DWV-A) and DWV-B [30], although other variants have been identified at much lower occurrence [31, 32]. DWV-B is the emerging genotype, currently replacing the previously prevailing DWV-A in Europe and North America in the 2010s [30]. DWV genotypes can vary in their molecular dynamics, with DWV-B titers outcompeting DWV-A when co-infecting [33], and intriguingly, only DWV-B appears to replicate within *Varroa* mites [34]. DWV-A and DWV-B share approximately 84% nucleotide sequence similarity [35, 36], and both genotypes, as well as their recombinants, have been found to negatively impact honey bee health [33, 37–42]. However, different studies have generated different outcomes with respect to the molecular dynamics and relative virulence of DWV-A versus -B– for example, McMahon and colleagues found that DWV-B was more virulent than DWV-A for adult bees ( [38], see also [43, 44]), whereas DWV-B was no different to [37] or lower than [33] DWV-A in virulence in pupae. Previous research has found that even within master variant classifications, virulence can differ [42]. DWV virulence, and the mechanism behind variant differences, therefore, require further investigation.

Viral infection in insects triggers a cascade of conserved pathways involved in the insect innate immune response [5, 45]. The honey bee immune response consists of many canonical antiviral pathways shared across insect species, although represented by fewer immune genes compared to *Drosophila melanogaster* [46]. These include the RNA interference (RNAi) pathway, which detects dsRNA produced during viral replication and uses these products for targeted degradation of viral transcripts [47]. Additionally, canonical pathways including Toll and Immune Deficiency (Imd) have been associated with host responses to DWV infection [47–49], as well as heat shock proteins [50], which are implicated in stress and infection [51, 52].

Beyond conserved immune pathways, honey bee molecular response to infection also consists of non-canonical immune genes. A key example is Vitellogenin, a yolk protein precursor that plays a role in important biological processes in honey bees, including behavioral maturation, nutrition, longevity, and immunity [53], and its downregulation is considered a biomarker of stress in adults [48, 54]. A meta-analysis of shared transcriptional responses to pathogens identified the down-regulation of metabolic pathway genes as a common response to infection, which may be adaptive to either host or pathogen [55]. Furthermore, exposure to other stressors, such as *Varroa* mites and pesticides, has been shown to affect a bee’s ability to mount an effective immune response [56–58], demonstrating the complexity and interconnectedness of biological pathways. As immune responses are resource intensive, and potentially damaging to the host, organisms must balance the trade-offs between immune response investment and other biological functions such as growth, development, and reproduction [59]. Such balancing of trade-offs has been observed in honey bees, including trade-offs between body mass and production of antiseptic enzymes involved in social immunity [60] and immune investment across sex and age stage [61].

Immune responses can also vary according to the genotype of the pathogen [62]. For example, strain-specific immune responses have been observed in mice with the rodent malaria parasite *Plasmodium yoelii* [63], respiratory syncytial virus in a mouse model [64], and with entomopathogenic fungal strains from the genus *Isaria* in the *Aedes aegypti* mosquito [65]. Norton et al. (2024) tested whether honey bees exhibited distinct responses to DWV variants, but did not find any expression differences in a subset of candidate immune genes [66]. However, since the honey bee molecular response to infection extends beyond canonical immune genes [55], a full transcriptomic analysis is necessary to identify viral-strain-specific immune responses.

To better understand the finer-scale differences in the molecular response to distinct viral genotypes, we sought to investigate both global immune gene expression to DWV infection, as well as responses specific to viral genotypes, and how this may relate to purported higher virulence of DWV-B over DWV-A. We evaluated responses of honey bee pupae, since this is the life stage where DWV infection is most common and detrimental. We conducted experimental infections with purified isolates of DWV-A and DWV-B, as well as a Mixed group of these two isolates, and assessed the transcriptional profile at 3 days post-infection compared to Controls. We hypothesized that across all DWV-positive groups, we would see shared responses to infection, as identified in a previous meta-analysis of honey bee transcriptional responses to DWV and other parasites [55]. Importantly, we sought to define viral-strain-specific transcriptomic response by comparing expression profiles between DWV-A, DWV-B, and Mixed infections, to gain further insights into the molecular mechanisms underpinning the emergence of DWV-B, and broadly how immune systems can respond to variable infectious agents.

## Methods

### Experimental infection samples and procedure

Experimental infections were conducted in Fall 2020, following protocols from Tehel et al. (2019) [37]. Two different colonies (thus representing distinct genotypes, C1 and C2) in the General Zoology apiary at Martin Luther University Halle-Wittenberg, Germany were used in this study. Colonies were visually inspected to ensure no to low *Varroa destructor* mite presence in the colony. Prior to infection studies, colonies were evaluated for viral infection via quantitative PCR (qPCR) to assess background levels of common bee viruses (DWV-A, DWV-B, acute bee paralysis virus (ABPV), black queen cell virus (BQCV), chronic bee paralysis virus (CBPV), slow bee paralysis virus (SBPV), and sacbrood virus (SBV)) using primers from [67]. Colonies were considered “virus free” by a threshold cycle of greater than 35. DWV-A and DWV-B inocula were those used in Tehel et al. (2019), with consensus sequences given in Figs. S3 and S4 of Tehel et al. (2019); inocula nucleotide similarity to two reference DWV genomes (DWV-A: NC_004830.2, DWV-B: NC_006494.1) was 97.4% and 99.3% respectively. Inocula were propagated in white-eyed honey bee worker pupae to obtain highly concentrated viral isolates (crude lysates), assessed for purity as described above, and quantified via standard curve (for detailed Methods, see Tehel et al., 2019; its Materials and Methods, Appendix A).

For experimental infections, honey bee workers at the white-eyed pupal stage were collected and kept within an incubator at 35 °C and 50% relative humidity until injection. Pupae that showed melanization, indicating injury during collection, were not used for experiments. DWV isolates used in this study were propagated from Tehel et al., (2019, see Sect. 2.2 for propagation methods). Briefly, crude isolates were derived from pupae that had been experimentally injected with 1 µL (10^4^ genome equivalents of DWV) of the original inoculum of Tehel et al. (2019) and then incubated at 35 °C for three days. Inoculum was injected laterally between its second and third abdominal tergites using a Hamilton syringe (32 gauge hypodermic needle, outer diameter: 0.235 mm), cleaning after each use and changing syringes between inocula to avoid contamination. After three days, crude viral isolates were prepared by homogenizing pupae in 0.5 M cold potassium phosphate buffer. For experimental infections, 1µL inoculum containing 10^3^ genome equivalents per µL of the propagated DWV-A, DWV-B, or an equal mix of DWV-A plus DWV-B (i.e. Mixed) was injected into each pupa. To measure the effect of the injection itself on gene expression, control bees (‘Control’) were injected with a virus-free inoculum prepared from uninfected bees of the same two colonies.

Injected pupae were kept in an incubator at 34.5 °C and 50% RH. Samples were collected at 3 days post injection (DPI), when DWV levels begin to plateau in the honey bee host [33]. To assess pupal eclosion rates as a metric for virulence, additional bees from three colonies (the two used in the gene expression study as well as one additional colony) were injected and allowed to develop past 3DPI (*n* = 48 per group).

### Virus screening from experimental infections

Collected samples were analyzed by quantitative PCR (qPCR) to confirm infection with DWV and confirm that there were no other infecting viruses, including ABPV, BQCV, CBPV, SBPV, and SBV, prior to submitting the samples for next-generation sequencing (Supplemental Table 26).

RNA isolation, cDNA synthesis, and qPCR were conducted as described in [37]. Briefly, RNA was isolated from whole bees using an RNeasy mini kit (Qiagen, Hilden, Germany). cDNA was synthesized from 800ng of RNA using oligo(dT)18 primers (Thermo Scientific, Waltham, MA, USA) and reverse transcriptase (M-MLV and Revertase, Promega, Mannheim, Germany) following the manufacturer’s protocol. cDNA was diluted 1:10x prior to qPCR reactions. qPCR was conducted using SYBRgreen Sensimix (Bioline, Luckenwalde, Germany) melt curve analysis to ensure the correct product had been amplified.

### Sequencing and analysis

RNA (*n* = 4 per group per colony) was submitted to GENEWIZ Germany (Leipzig, Germany) for library preparation sequencing on the Illumina NovaSeq platform, resulting in 150 nucleotide pair-end stranded mRNA reads. Total reads ranged from 28 to 71 million per sample. Reads were assessed for quality with FastQC (version v0.11.9) [68], and quality trimmed with Trimmomatic (version 0.39, option SLIDINGWINDOW:4:30) [69], retaining on average 96.3% of reads across all samples.

Reads were aligned with Hisat 2 (version 2.1.0) to the DWV reference genomes from NCBI (DWV-A: NC_004830.2, DWV-B: NC_006494.1) to calculate the percentage of DWV reads in each sample (Supplemental Table 1). One-way analysis of variance (ANOVA) was used to compare the sum of DWV-aligning reads in R (version 3.6.3), using the stats package.

Kallisto (version 0.46.2) [70] was used for pseudo-alignment and estimation of transcript abundance on the *Apis mellifera* genome assembly (HAv3.1) from NCBI (GCF_003254395.2). Transcript estimates were compiled in R (version 3.6.3), and summed by gene using tximport (version 1.14.2, Supplemental Table 2) [71], as in [72].

Differentially expressed genes (DEGs) across groups (Control (C), DWV-A (A), DWV-B (B), and Mixed (M)) were assessed using Limma-Voom (version 3.42.2) [73, 74]. Low count genes, representing less than 1 count per million (cpm) across two samples were removed, resulting in a gene list of 10,375, and were normalized to the trimmed mean of M-values (TMM). Mean-variance relationship of the log2 counts per million (CPM) estimates was used for variance stabilization calculation, which was then used for linear model fitting with empirical Bayes smoothing of standard errors [75]. Benjamini-Hochberg (BH) correction for multiple testing was used throughout, and DEGs were assigned across groups at a threshold of adjusted *p*-value < 0.05, both with and without a log2 fold change (LFC) expression threshold (LFC = 1 or 0, respectively).

DEG overlaps were made with SuperExactTest (version 1.1.0) [76] to calculate significant overlaps with BH correction. For Gene Ontology (GO) enrichment analysis, DEGs were converted to *Drosophila melanogaster* protein-coding orthologs, when possible, then uploaded to the GOrilla online database for enrichment analysis against a global ortholog list of 7,419 genes [77]. Raw sequence reads can be found on the NCBI SRA database (Bioproject: PRJNA1223014).

## Results

### Pupal survival rates across DWV groups

Bees that were allowed to develop past 3DPI were assessed for adult eclosion rates as a metric for virulence (Supplemental Table 25). At approximately 7 days post-injection, nearly all injected Controls eclosed successfully (47/48 successfully eclosed, 98%). All DWV inocula were found to be highly virulent compared to Controls (Pearson’s χ2 test, *p*-value < 0.001): only 6.3% of DWV-B injected bees survived to eclosion, and no bees survived to eclosion in the DWV-A and Mixed groups. While only bees exposed to DWV-B were able to survive to eclosion (2/48), this was not significantly different to the DWV-A and Mixed groups (0/48) (Pearson’s χ2 test, *p*-value > 0.05).

### Confirmation of infection status

**Fig. 1:**
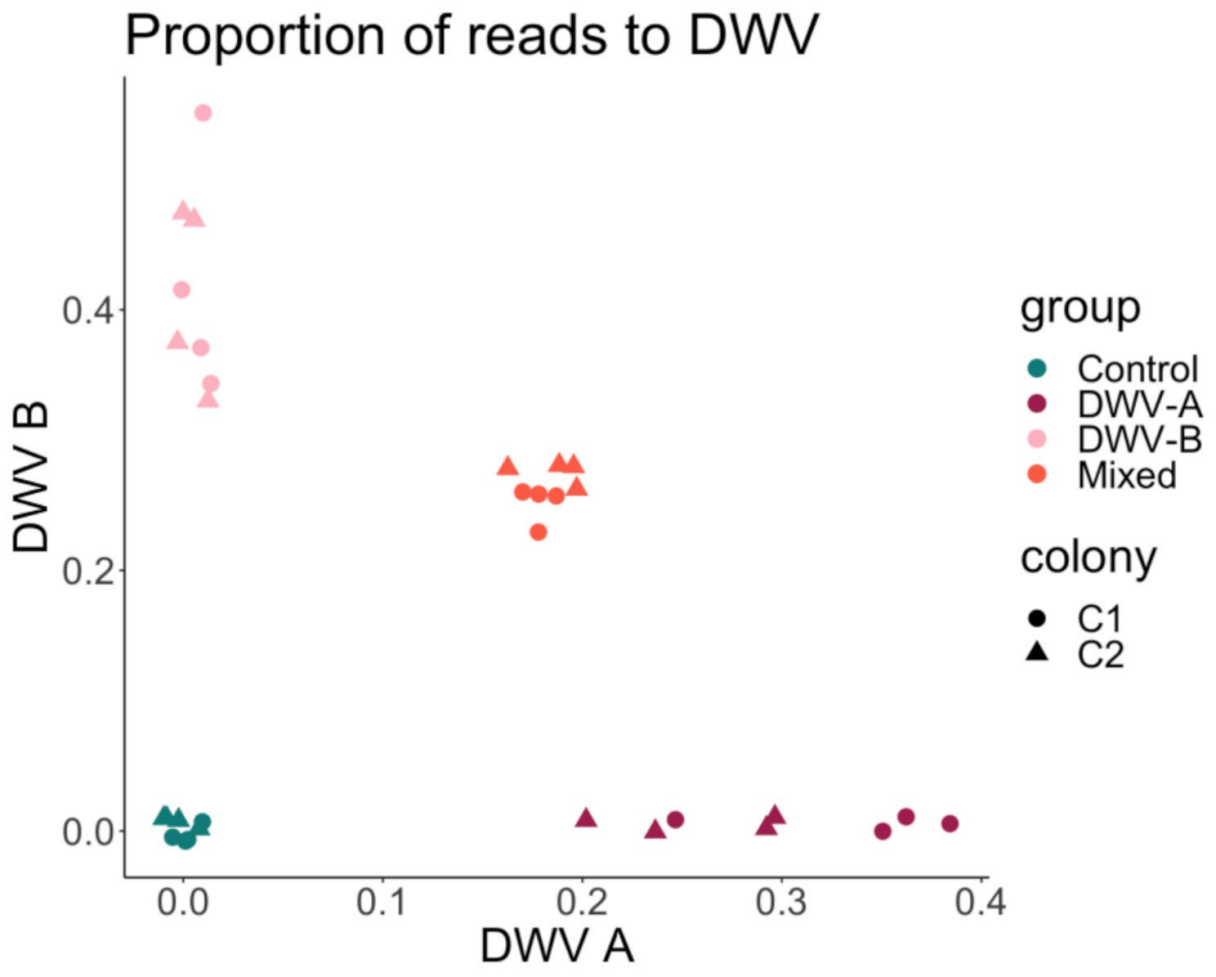
Proportion of DWV reads across groups confirms infection status and little to no background DWV contamination in Controls. Proportions were calculated from the number of reads that successfully aligned to the DWV-A or DWV-B genome over the total count of reads for each sample. Color indicates group (Control (C) = teal, DWV-A (A) = maroon, DWV-B (B) = pink, and Mixed (M) = orange) and shape indicates the sample’s colony of origin (C1 or C2), *n* = 4 per colony per group

Quality trimmed reads were aligned to the two reference DWV genomes used in this study (DWV-A: NC_004830.2, DWV-B: NC_006494.1) to confirm infection and measure possible contamination (Supplemental Table 1). The proportion of total reads that aligned to either DWV-A and DWV-B reads in DWV + groups ranged from 17 to 56% of total trimmed reads, whereas the proportion of DWV reads in Control samples was less than 0.3% of the total reads (Fig. 1). The sum of DWV reads was significantly lower in DWV-A groups compared to DWV-B and Mixed (One-way ANOVA, *p*-value < 0.001), but was not significantly different between DWV-B and Mixed (One-way ANOVA, *p*-value = 0.76).

### Evaluation of transcriptomes

On average, we obtained approximately 22.3 million pseudo-alignments per individual pupa to the honey bee reference genome (Amel_HAv3.1) (Supplemental Table 2). Multidimensional scaling of log2 fold change (LFC) expression profiles demonstrates clustering by group with high overlap of DWV + groups, particularly DWV-A and Mixed-infection groups (Supplemental Fig. 1).

The number of DEGs across groups (with and without log2 fold change (LFC) cut-off) is provided in Table 1. Genes that survived the LFC threshold cut-off of 1 show a two-fold or greater difference in expression. Of the total DEGs, 2,906 genes were differentially expressed between DWV-A and Control, and 3,827 genes were differentially expressed between Mixed and Control, but only 189 DEGs between DWV-B and Control. 16.4% and 14.4% of DEGs survived the LFC threshold in Control vs. DWV-A and Control vs. Mixed infection, respectively, resulting in 476 and 550 DEGs. Though fewer genes were differentially expressed in bees with DWV-B infections compared to Control, a greater percentage (44.4%) of these were above the LFC threshold, resulting in 84 DEGs after LFC filtering (Supplemental Tables 3–19). Overall, these results demonstrate that DWV-A– alone or in a Mixed infection– triggers a substantial host immune response, while DWV-B infection has a much smaller impact on host gene expression.

**Table 1:** Differentially expressed genes across treatment groups, with and without log fold change (LFC) cutoffs, adjusted *p*-value < 0.05

	No LFC threshold		LFC threshold: 1	
	Upregulated	Downregulated	Upregulated	Downregulated
DWV-A vs. Control	1454	1452	374	102
DWV-B VS Control	134	55	80	4
Mixed vs. Control	1883	1944	437	113
DWV-A vs. DWV-B	139	153	57	24
DWV-A vs. Mixed	0	0	0	0
DWV-B vs. Mixed	327	523	26	105

*Out of 10,375 genes

Within DWV + groups, 84 and 131 DEGs were found when comparing infection of DWV-B to DWV-A and Mixed at the LFC threshold, respectively, but no significant differentially expressed genes were observed between DWV-A and Mixed infections even without implementing the LFC threshold. These results demonstrate that DWV-A results in different– and greater– gene expression responses than DWV-B, and DWV-A dominates the gene expression response in Mixed infections.

**Fig. 2:**
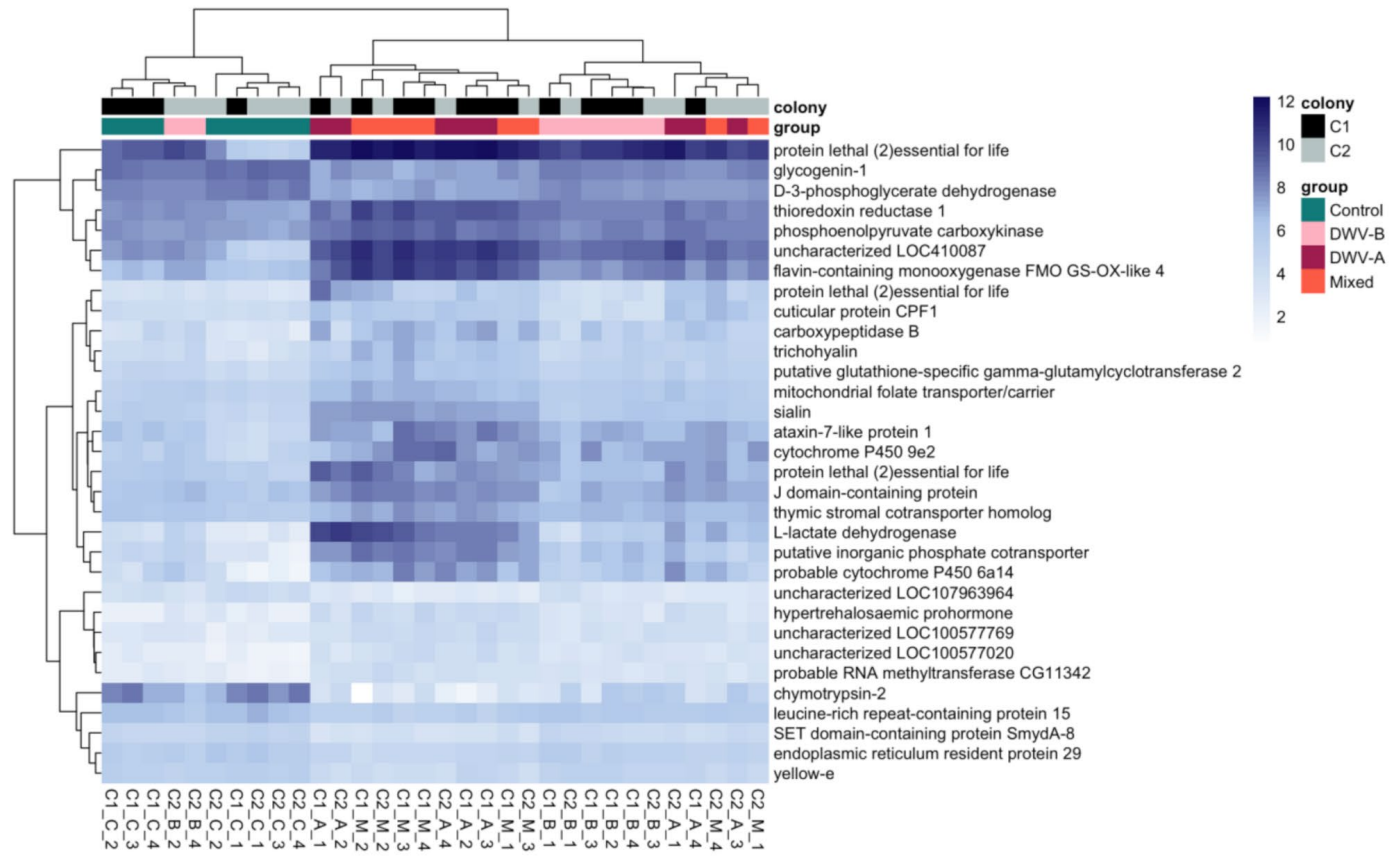
Expression pattern of the top 5% of DEGs. Clustering depicts similar expression of DWV-A (A, maroon) and Mixed (M, orange) expression profiles, while DWV-B (B, pink) clusters with both Control (C, teal) and other DWV groups, irrespective of sample’s colony of origin (black or gray). Genes (listed on right) were also clustered based on their expression similarity. Color scale depicts lower (gray) to higher (blue) log2 counts per million

For further examination of DEGs across groups, we focused on genes that survived the fold change cut-off (LFC > 1). The expression pattern for the top 5% of significantly expressed genes based on adjusted *p*-value (32/637) genes can be found in Fig. 2. Similar to the multidimensional scaling analysis, DWV-A and Mixed groups cluster together, with DWV-B interspersed with Control and the other DWV groups.

### Genes consistently differentially regulated in DWV-A, DWV-B, and mixed infections

**Fig. 3:**
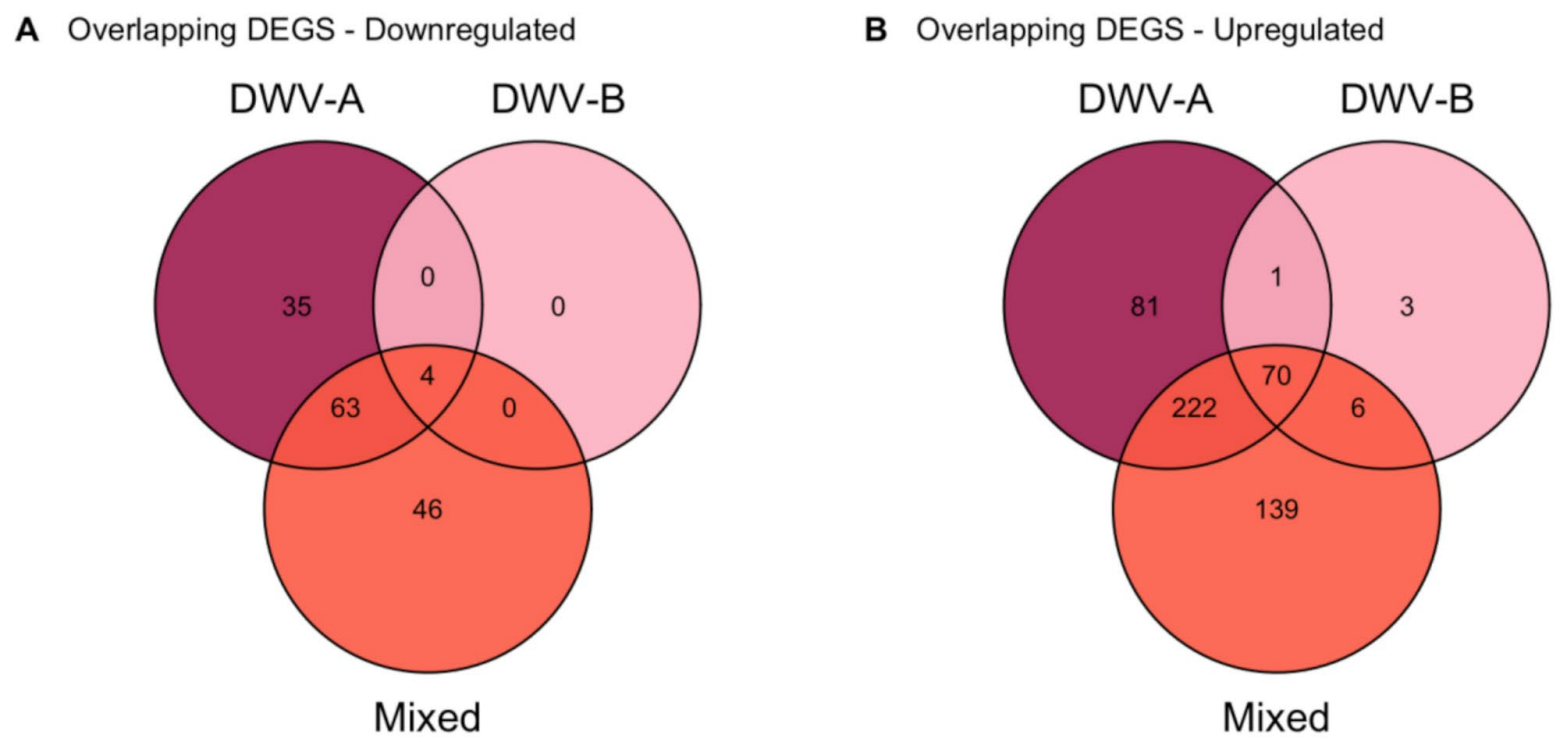
DEG overlaps across DWV + groups. Overall, we see a higher overlap between DWV-A and Mixed infection in Downregulated (**A**) and Upregulated (**B**) genes compared to Control

To establish the general immune response to DWV in our experimental infections, we compared the intersection between the genes differentially transcribed in the DWV-A, DWV-B, and Mixed groups compared to Controls. There was significant overlap within our DWV + groups of genes upregulated or downregulated compared to Controls (Fig. 3, Supplemental Table 22). When considering genes that were consistently regulated in DWV-A, DWV-B, and Mixed, we found 4 genes were downregulated compared to Control (hypergeometric test, fold enrichment [FE] = 35.9, *p* < 0.001) and 70 genes were upregulated with DWV infection across all three groups (hypergeometric test, FE = 35.1, *p* < 0.001) (Fig. 3). Genes that were consistently differentially expressed in the three DWV-infected groups versus the Control group largely did not correspond with the canonical immune response genes (Supplemental Tables 14–15).

The top 4 genes downregulated in DWV infection across all DWV + groups included the serine protease chymotrypsin-2 (LOC409626), the feline leukemia virus subgroup C receptor-related protein 2 (LOC107965219), as well as two uncharacterized genes (LOC102653658 and LOC102654085). Of the 70 genes upregulated in DWV versus Control, the top 2 genes with the greatest fold change difference included two uncharacterized protein-coding genes (LOC100576152 and LOC726094). While no match was found for LOC100576152 on the Alphafold protein structure database [78], the predicted structure for LOC726094 is a low-confidence match with Osiris genes of other insects, and Osiris genes have previously been associated with bee immunity [79]. Other highly upregulated genes in the DWV + group included chitinase-3-like protein 1 (LOC100577156), cytochrome P450 6AS5(Cyp6as5 - LOC409677), glycine N-methyltransferase (LOC552832) and protein lethal (2) essential for life (LOC724367).

### Genes showing virus-strain-specific responses to infection

**Fig. 4:**
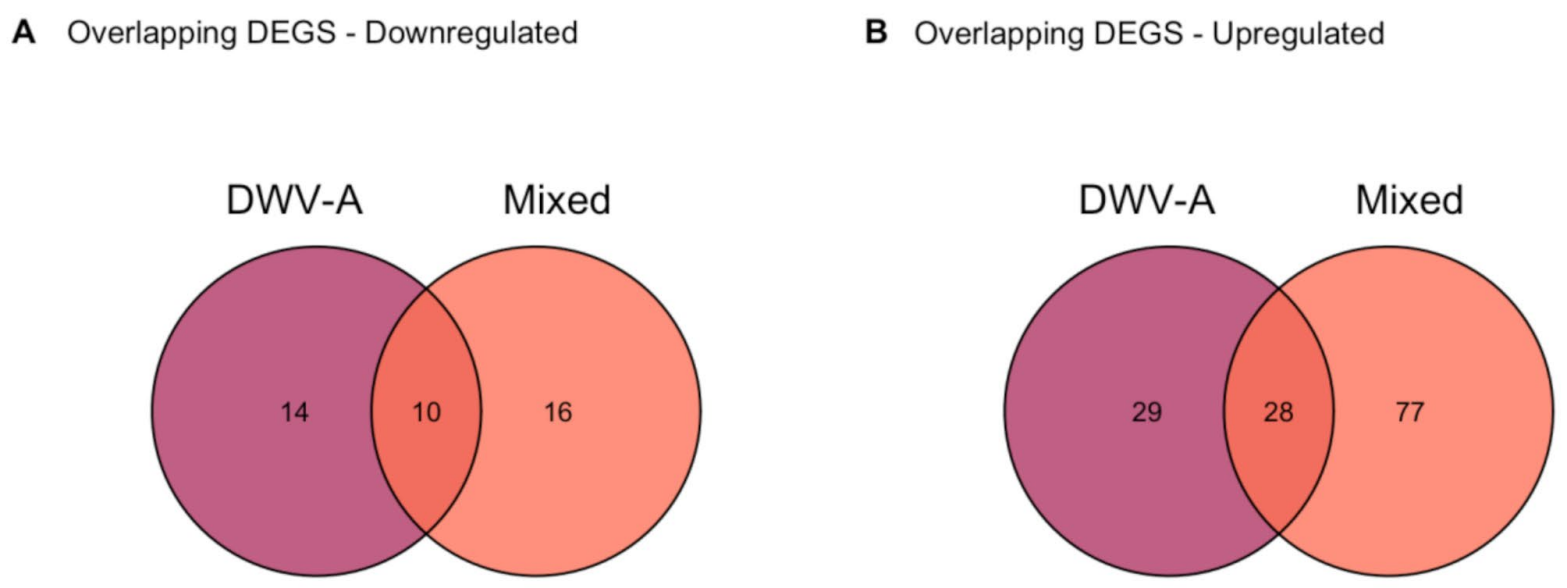
DEG overlaps between DWV-A and Mixed groups compared to DWV-B. Ten genes were consistently downregulated in both DWV-A and Mixed infections relative to B (**A**), and twenty-eight genes were consistently upregulated in DWV-A and Mixed infections compared to DWV-B (**B**)

After establishing the immune response shared across all DWV + groups compared to Controls, we then sought to examine the finer-scale transcriptional differences between DWV-A, DWV-B, and Mixed infections. To examine differential expression across DWV + strains, we compared the DEG overlaps between DWV-A, DWV-B, and Mixed infections. As there were no DEGs when comparing DWV-A and Mixed infections (Table 1), we used DWV-B infection as our baseline expression and compared DEGs in DWV-A and in Mixed compared to DWV-B. We found 10 overlapping genes downregulated (hypergeometric test, FE = 166.3, *p* < 0.001) and 28 genes upregulated (hypergeometric test, FE = 48.5, *p* < 0.001) in DWV-A and Mixed infection compared to DWV-B (Fig. 4).

Of the 10 downregulated genes shared across DWV-A and Mixed infection compared to DWV-B, we find two leucine-rich repeat proteins, LOC100577598 and LOC100576903, which are associated with sensing pathogen-associated molecular patterns (PAMPs), as well as nicotinic acetylcholine receptor alpha9 subunit (nAChRa9, LOC411303), lysosomal alpha-mannosidase (LOC552249), and multiple uncharacterized genes (Supplemental Table 18).

Of the 28 genes upregulated in DWV-A and Mixed infection compared to DWV-B, the gene with the highest fold change difference was the glycolysis gene l-lactate dehydrogenase (LOC411188). The other top upregulated genes included proline-rich protein 36-like (LOC102655429), two heat shock proteins (protein lethal (2) essential for life (LOC412197 and LOC724449)), and two cuticular proteins (CPF1 and CPR2) (Supplemental Table 19).

**Fig. 5:**
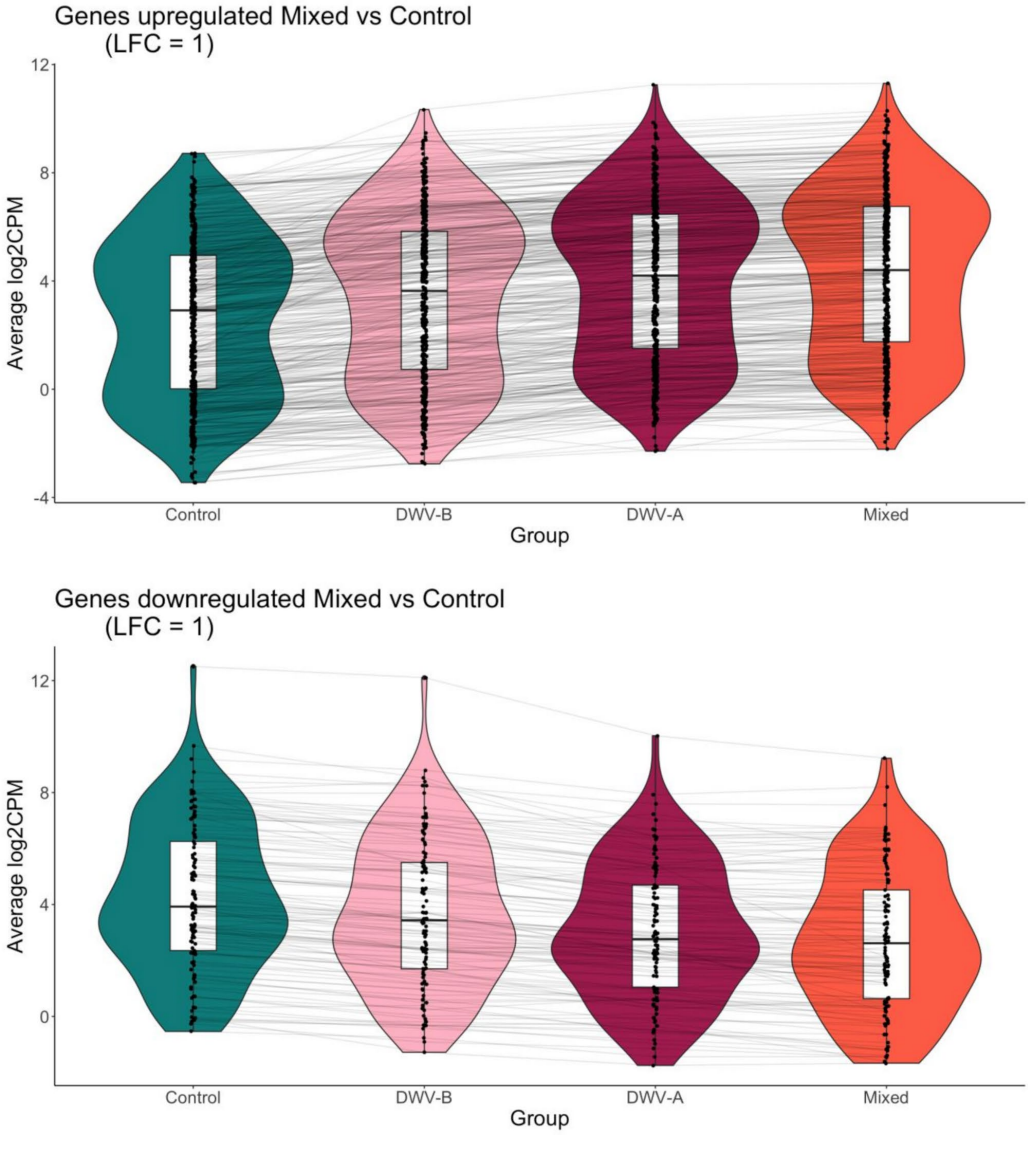
Average Log2 counts per million (CPM) of the 437 genes upregulated in Mixed versus Control (above) and 113 genes downregulated in Mixed versus control (below). Each dot represents a different DEG, and lines connect the same DEG across groups (Control, DWV-B (**B**), DWV-A (**A**), and Mixed). Average was calculated over 4 samples per group and 2 colonies: *n* = 8 per gene per group

While DWV-A and Mixed infections had the highest number of DEGs compared to Control, often the expression of these genes in the DWV-B group trended towards differential expression compared to Control, but did not always satisfy the LFC threshold requirements (Fig. 5). Thus, the expression patterns in DWV-B infected bees seem to be similar to those of the DWV-A and Mixed infection bees, but just at a lower level.

### Differential regulation of immune response genes

DEGs from DWV + groups did not significantly overlap with honey bee immune pathways (hypergeometric test, *p* > 0.1) [46], but a subset of canonical immune genes were differentially expressed in response to infection (Table 2, Supplemental Table 20). The antimicrobial peptides (AMPs) abaecin (LOC406144) and hymenoptaecin (LOC406142) were upregulated across all three DWV groups. DWV-A and Mixed infections also upregulated toll-like receptor 6 (LOC410229), defensin 1 (Def1), and apoptosis-associated gene caspase 3 (LOC411381) compared to Controls. Two toll-pathway genes were also found to be downregulated in DWV-A and Mixed infection compared to Control: lysozyme 2 (LOC724899) and proclotting enzyme (PPOAct - LOC726126) (Table 2).

**Table 2:** Differentially expressed canonical immune genes (Evans et al., 2006, LFC > 1) in DWV + groups. Bold squares indicate significance at the LFC threshold (< 0.05) after *p*-value adjustment

NCBI_ID	pathway	Gene	LFC				
			AvC	BvC	MvC	BvA	BvM
411,381	Apoptosis	caspase-3 (LOC411381)	**3.4**	2.1	**3.7**	-1.3	**-1.6**
725,154	Toll	serine protease snake (LOC725154)	**1.0**	0.6	**1.1**	-0.5	-0.6
412,484	Toll	peptidoglycan recognition protein S2 (Pgrp-s2)	0.1	0.4	**1.2**	0.4	-0.8
408,317	Toll	uncharacterized LOC408317– spätzle 6 (LOC408317)	**1.1**	**1.2**	**1.5**	0.1	-0.3
410,229	Toll	Toll-like receptor 6 (LOC410229)	**1.1**	0.3	**1.3**	-0.8	**-1.0**
406,144	Toll	abaecin (LOC406144)	**1.6**	**2.2**	**2.3**	0.6	0.0
406,143	Toll	defensin 1 (Def1)	**2.9**	2.8	**3.4**	-0.1	-0.5
406,142	Toll	hymenoptaecin (LOC406142)	**1.7**	**3.0**	**1.3**	1.4	1.7
724,899	Toll	lysozyme (LOC724899)	**-1.8**	-0.7	**-1.5**	1.1	0.8
726,126	Toll	proclotting enzyme (LOC726126)	**-1.2**	-0.7	-1.0	0.5	0.3

Beyond canonical immune pathways, genes upregulated in DWV + groups had a significant degree of overlap with putative serine protease genes (hypergeometric test, *p* < 0.05) [80], particularly in DWV-A and Mixed infections (Supplemental Table 21), with one exception, chymotrypsin 2, which was significantly upregulated in Controls. Additionally, genes upregulated during infection significantly overlapped with DEGs from the Doublet et al., 2017 meta-analysis of conserved molecular responses to pathogens (hypergeometric test, *p* < 0.005) (Table 3). The highest fold enrichment of our upregulated genes were also the genes found upregulated in the Doublet et al. dataset, although we do also see significant overlap in the genes downregulated across the Doublet dataset, as well as Doublet DEGs overall (Table 3).

**Table 3:** Degree of overlap with DEGs upregulated in infection compared to the Doublet et al. (2017) meta-analysis DEG lists

Group		Observed overlap	Fold Enrichment	Adjusted *p*-value
DWV-A vs. Control (374)	DEGS	43	3.9	1.9E-13
	UP	17	8.3	1.1E-10
	DOWN	16	4.0	1.3E-05
DWV-B vs. Control (80)	DEGS	17	7.2	1.5E-09
	UP	8	18.2	1.1E-07
	DOWN	5	5.9	0.006
Mixed vs. Control (437)	DEGS	48	3.7	2.9E-14
	UP	18	7.5	1.2E-10
	DOWN	17	3.7	2.1E-05
DWV-A vs. DWV-B (57)	DEGS	7	4.2	0.005
	UP	3	9.6	0.014
	DOWN	1	1.7	n.s.
DWV-B VS MIXed (105)	DEGS	15	4.8	3.2E-06
	UP	6	10.4	1.2E-04
	DOWN	7	6.3	6.1E-04

Number of DEGs per Group listed in parentheses. Number of DEGs per Doublet et al. group: Total DEGS: 307, UP: 57, DOWN: 110

### GO analysis

Overall, functional enrichment with DEGs with LFC > 1 did not result in many GOs after FDR adjustment. DWV-A and Mixed infections compared to Controls were significantly enriched for GOs relating to serine protease activity, and all three DWV groups were significantly enriched for extracellular space components (Supplemental Table 23). No significant GOs were found for genes upregulated in Controls compared to any DWV group. When examining GOs with all DEGs (LFC threshold: 0), we observed many more significant GOs after FDR correction across DWV + groups. Of note, of the genes downregulated in DWV infection, we see enrichment for GOs related to mitochondrial translation (Supplemental Table 24).

## Discussion

Here, we examined whether infection by different variants of deformed wing virus (DWV) resulted in unique transcriptional profiles in the honey bee (*Apis mellifera*). We performed experimental infections with pure DWV-A and DWV-B isolates, as well as a Mixed infection containing both variants, in white-eyed pupae, mimicking the start of the honey bee developmental stage targeted by reproductive *Varroa* parasites and associated DWV infection. We then assessed whole-body transcriptomes 3 days post-injection. We found that DWV infection across all groups did result in a significant change in gene expression compared to Controls. Moreover, there were distinct transcriptional profiles between DWV-B infection compared to the other DWV + groups. Significantly differentially expressed genes (DEGs) included a subset of canonical immune genes, as well as key energetics and metabolic factors, tying DWV variant epidemiology and the emergence of DWV-B to honey bee immunometabolic responses to viral infections.

DWV-B infection versus Control resulted in fewer differentially regulated genes compared to DWV-A and Mixed, although expression of many DEGs from DWV-A and Mixed appear to be at somewhat intermediate expression in DWV-B. For example, alpha-mannosidase (LOC552249), a gene implicated in arbovirus infections and *Wolbachia*-mediated pathogen blocking mosquitoes [81, 82], is significantly downregulated in DWV-A and Mixed compared to Control (LFC = -2.1 and − 1.9, respectively); but decreased expression of this gene did not meet the log fold change cutoff in DWV-B compared to Control (LFC = -0.79). L-lactate dehydrogenase was significantly upregulated in DWV-A and Mixed infection versus Control (LFC = 4.8 and 5, respectively), but also in DWV-B infection compared to Control, just to a lesser extent (LFC = 1.7). This indicates that the viral strain-specific transcriptional response does not result in different suites of genes, per se, but instead manifests through the intensity of activation of immune genes. If the immune response itself produces some immunopathological side effects, this may provide one mechanism behind virulence differences across viral strains [37, 38, 83]. Indeed, we did see slightly higher pupal survival in the DWV-B group, yet DWV titers appear to be higher in DWV-B infected samples than in the DWV-A group. Rather than resisting the virus, hosts may instead minimize the negative impacts of infection, for example, by decreasing their immune response, thereby minimizing immunopathology-induced damage, demonstrating a tolerance immune strategy to infection with DWV-B. Alternatively, DWV-B may be able to evade the host’s immune response, leading to higher viral titer in pupae (this study) and adults [38] and higher virulence in adults. Further investigation is needed to disentangle the pathogenic effects of viral replication versus immune response, and how this relates to disease tolerance and resistance.

The mechanism driving differential response to DWV strains, and how these genes affect infection, is not obvious. Gene expression seemed not to be driven by titer alone, as DWV-B on average has a higher proportion of DWV-aligning reads compared to DWV-A and a similar abundance of DWV reads to the Mixed group (Fig. 1), yet had a more similar expression profile to Control, with fewer DEGs compared to DWV-A and to Mixed (Table 1). Genes differentially expressed after exposure could facilitate viral replication or act as immune effectors. Indeed, a number of pro- and anti-viral factors have been identified in *Drosophila melanogaster* [84]. Expression differences may be driven by the honey bee host’s detection of particular genotypes within a viral population, tailoring the immune response to specific strains, versus virus-derived factors directly altering gene expression. We observed no significant difference in the molecular response of DWV-A and Mixed infections (versus a hybrid transcriptional profile to DWV-A and DWV-B), meaning the immune system may be triggered by the detection of DWV genotype A. It is possible that DWV-B may be able to evade the host immune detection, resulting in more rapid proliferation [26, 33, 38], and a lessened effect on the transcriptional profile compared to infection by DWV-A that we observe here. If the transcriptional differences were driven by DWV-B suppressing the honey bee immune response, we would have expected partial suppression of immune activation in Mixed infections: instead, we saw the most DEGs in the Mixed group when compared to Control, suggestive of immune evasion by DWV-B.

While we found differences across DWV strains, we also observed overlaps of DEGs across all DWV groups compared to Control, indicative of core transcriptomic responses to DWV infection. This core response included upregulation of AMPs and other Toll pathway genes, similar to previous DWV studies [39, 48, 85–88]. We also observed upregulation of serine protease genes, also upregulated in IAPV infection [89] and broadly involved in innate immunity as well as development and digestion [80], and upregulation of heat shock proteins lethal (2) essential for life [47, 50]. Furthermore, we saw a significant overlap with the genes identified in a meta-analysis of immune response [55]. Identification of these immune response pathways in our study and across different populations exposed to pathogens provides further evidence for a conserved, global antiviral response in honey bees, consisting of both canonical and non-canonical immune genes.

Downregulated genes within the core response to DWV infection were limited to only 4 genes shared across all DWV groups, with two representing currently uncharacterized genes. Chymotrypsin-2 was downregulated in DWV infection, and also differentially expressed in other studies [47, 49, 89]. Chymotrypsin-2 may act to regulate the activated Toll pathway [90] or alternatively represents a less costly, constitutive immune effector, which is switched off when specific immune factors like AMPs are expressed. Downregulation of feline leukemia virus subgroup C receptor-related protein 2 appeared to be unique to this study, and may be involved in regulation of heme transport [91], which may in turn affect cytochrome P450 activity during infection. Our identification of this gene may be due to recent genome annotation updates [92], or alternatively, it may be a result of host genotypic variation in immune gene expression [93].

We also observed intriguing expression patterns of metabolism genes in the molecular response to DWV. A key gene upregulated across DWV infection was l-lactate dehydrogenase (LOC411188). This gene was also designated a top DEG and “hub” gene (i.e. high co-expression with other DEGs) in a meta-analysis of honey bee pathogen response [55]. Moreover, it was upregulated in bees with fatal IAPV infections [94], and upregulated in low-aggression bees with “sick-like” molecular signatures [95]. Lactate dehydrogenase catalyzes the reversible reaction of pyruvate to lactate, and its upregulation, such as in cancer cells, is associated with aerobic glycolysis (AG), a.k.a. the “Warburg Effect” [96]. During AG, glucose is metabolized to lactate, rather than undergoing oxidative phosphorylation (OXPHOS), despite the presence of sufficient oxygen. While AG generates ATP much less efficiently than OXPHOS (approximately 2–4 mol ATP vs approximately 36 mol ATP per mol glucose), it is able to rapidly produce ATP, NAD+, and alter concentrations of metabolites that may be beneficial during infection. In addition to increased lactate dehydrogenase expression, DWV + groups exhibited other characteristics associated with AG, including higher expression of pentose phosphate pathway components, and are underrepresented for GOs related to mitochondrial translation and electron transport chain, although this enrichment was only measured when assessing total DEGs (LFC threshold = 0).

Aerobic glycolysis has been implicated in a suite of infections across species (reviewed in [97]) including invertebrates [98–100]. A shift from OXPHOS to AG has also been observed in honey bee aggression behavior [101, 102], potentially leading to an increase in production of glutamate, a putative neurotransmitter and/or glycolysis modulator in the honey bee brain [103]. Additionally, AG during infection may promote rapid honey bee cell proliferation [96], and may explain why there is more rapid development in DWV-infected bees [42, 104]. While glucose consumption’s direct impact on viral disease in bees has not been characterized, a recent study by Chen et al. (2021) found increased glucose and ATP levels in the bodies of DWV-infected bees [105], and virus-infected bees are highly responsive to sucrose [106, 107]. However, it is not known whether this putative AG resulting from DWV infection is more beneficial for viral replication [97] or the honey bee host mounting a rapid immune response [108, 109].

## Conclusions

Viral genotype can shape disease outcomes, and the mechanism behind this effect in DWV-infected bees requires further investigation. The next steps to identify causative links between viral strain, honey bee immune response, and infection outcomes, particularly related to immunometabolism and potential host-damaging effects of immunity, will shed light on these complex host-microbe interactions. Functional characterization of putative genes expressed in infection, including numerous ‘uncharacterized’ genes within the honey bee genome, is necessary to provide more insight into honey bee immune dynamics. The interplay between metabolism and immunity during DWV infection can be further explored through metabolomics as well as gene expression manipulations through knockouts and knockdowns [110, 111]. How viral genotype affects immune response can be investigated with the recent development of DWV infectious clones [112], and overall the complex interaction between virus genotypes and hosts can be explored through large-scale, population genetics approaches [113]. As honey bee disease outcomes have already been associated with numerous biotic and abiotic factors [26, 56, 114–117], this expanding, more holistic understanding of honey bee disease is crucial to investigating virus evolution and supporting global pollinator health. Overall, this study provides the first evidence for strain-specific immune responses to DWV infection, and integrates these findings into the broader domain of insect immunity and host-pathogen dynamics.

## Electronic supplementary material

Below is the link to the electronic supplementary material.

Supplementary Material 1

Supplementary Material 2

## Data Availability

Supplementary data is provided in the Supplementary Material. Raw sequence reads can be found on the NCBI SRA database (Bioproject: PRJNA1223014).

## Abbreviations

DWV: Deformed wing virus
RNAi: RNA interference
Imd: Immune Deficiency
qPCR: Quantitative PCR
DPI: Days post injection
DEGs: Differentially expressed genes
TMM: Trimmed mean of M-values
CPM: Counts per million
BH: Benjamini-Hochberg
LFC: Log(2)fold change
GO: Gene Ontology
ANOVA: Analysis of variance
MDS: Multidimensional scaling
FE: Fold enrichment
AMP: Antimicrobial peptide
AG: Aerobic glycolysis
OXPHOS: Oxidative phosphorylation
