# Supplementary material for: The intensity of the transcriptional response varies across infection with distinct viral strains in an insect host
Source: BMC Genomics. 2025 Feb 21;26:175. doi: 10.1186/s12864-025-11365-8 (PMC11846320; doi:10.1186/s12864-025-11365-8)
Supplement: Supplementary file 1 — Supplementary Material 1 [file 12864_2025_11365_MOESM1_ESM.docx]

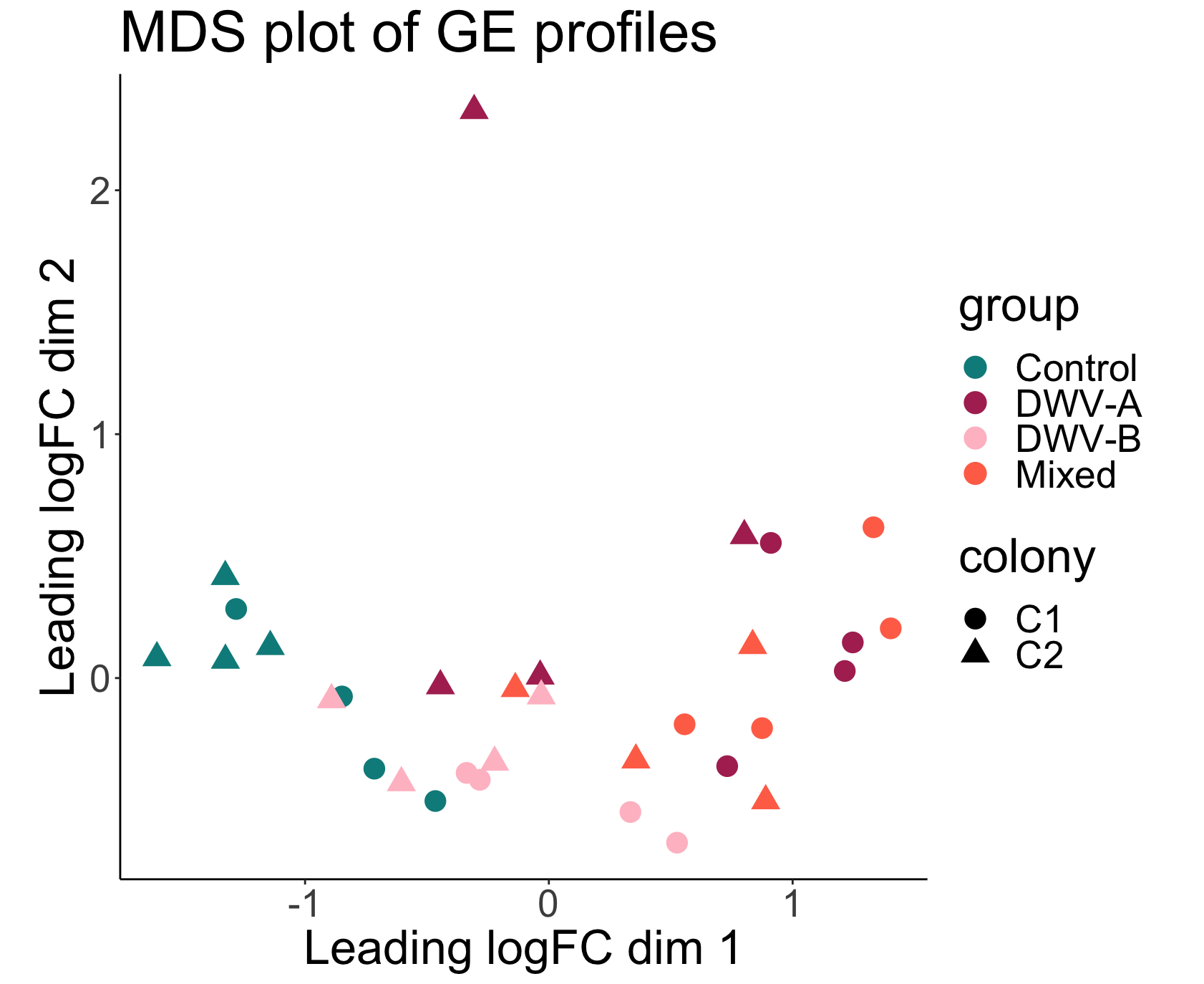


**Supplemental Figure 1.** Multidimensional scaling (MDS) plot of normalized log2 fold change expression across samples. Dimension 1 depicts a high divergence log2-fold-change between the Control and DWV-A/Mixed-infection groups, with DWV-B overlapping in the middle. Color indicates group (Control (C) = teal, DWV-A (A) = maroon, DWV-B (B) = pink, and Mixed (M) = orange) and shape indicates the sample’s colony of origin (C1 or C2).
